# Spatial domain identification method based on multi-view graph convolutional network and contrastive learning

**DOI:** 10.1371/journal.pcbi.1013369

**Published:** 2025-10-17

**Authors:** Xikeng Liang, Shutong Xiao, Lu Ba, Yuhui Feng, Zhicheng Ma, Fatima Adilova, Jing Qi, Shuilin Jin

**Affiliations:** 1 School of Mathematics, Harbin Institute of Technology, Harbin, China; 2 V.I. Romanovsky Institute of Mathematics, Uzbekistan Academy of Sciences, Tashkent, Uzbekistan; Carnegie Mellon University, UNITED STATES OF AMERICA

## Abstract

Spatial transcriptomics is a rapidly developing field of single-cell genomics that quantitatively measures gene expression while providing spatial information within tissues. A key challenge in spatial transcriptomics is identifying spatially structured domains, which involves analyzing transcriptomic data to find clusters of cells with similar expression patterns and their spatial distribution. To address these challenges, we propose a novel deep-learning method called DMGCN for domain identification. The process begins with preprocessing that constructs two types of graphs: a spatial graph based on Euclidean distance and a feature graph based on Cosine distance. These graphs represent spatial positions and gene expressions, respectively. The embeddings of both graphs are generated using a multi-view graph convolutional encoder with an attention mechanism, enabling separate and co-convolution of the graphs, as well as corrupted feature convolution for contrastive learning. Finally, a fully connected network (FCN) decoder is employed to generate domain labels and reconstruct gene expressions for downstream analysis. Experimental results demonstrate that DMGCN consistently outperforms state-of-the-art methods in various tasks, including spatial clustering, trajectory inference, and gene expression broadcasting.

## Author summary

Spatial transcriptomics technology not only provides high-resolution gene expression data but also completely preserves the spatial location information of each sequencing spot in tissue sections, offering an unprecedented multi-dimensional perspective for in-depth exploration of tissue heterogeneity and dissection of cellular microenvironments and functional partitions. Similar to the cell clustering task in single-cell data analysis, one of the core challenges in spatial transcriptomics data analysis is spatial domain identification—using algorithms to cluster sequencing spots with adjacent spatial locations and similar gene expression patterns into biologically meaningful functional regions. Although existing methods can achieve preliminary spatial domain partitioning, accurately capturing global semantic associations and balancing the modeling capabilities of local features and global structures remain critical scientific challenges when faced with non-linear gene expression patterns and spatial distribution relationships in complex tissues. Here, we propose a novel algorithmic framework that integrates multi-view graph convolutional networks and contrastive learning. Results across multiple datasets generated by different technologies demonstrate that our method significantly enhances the accuracy and biological interpretability of spatial domain identification.

## Introduction

Spatial Transcriptomics (ST) is a rapidly evolving biotechnology in recent years that allows researchers to observe gene expression in individual cells or spots, and access the specific spatial location of these cells in a tissue. Spatial information significantly enhances our understanding of how cellular gene expression is influenced by environmental factors, making it crucial for elucidating complex biological processes and the underlying mechanisms of diseases. Various platforms for spatially resolved transcriptomics have gained prominence, including in situ hybridization (ISH) technologies such as seqFISH [1,2], seqFISH+ [3], and MERFISH [4,5], as well as in situ capturing techniques like HDST [6], ST [7], Slide-seq [8], and 10x Genomics Visium [9]. Additionally, numerous databases such as stSNV [10] and STOmicsDB [11] have curated spatial omics data, providing valuable resources for studying tissue heterogeneity and biological mechanisms to support in-depth exploration of cellular interactions and disease mechanisms in spatial contexts.

One of the most important topics in spatial transcriptomics is the identification of spatial structural domains (i.e., regions that are spatially aligned in gene expression and histology). This task involves analyzing spatial transcriptomic data to identify clusters of cells with similar expression patterns and their spatial distribution in tissues.The core challenge of spatial domain recognition lies in integrating gene expression with spatial information to reveal cellular interactions. Existing methods leverage graph-based frameworks and deep learning to address this: DeepST [12] fuses morphological features from images with spatial and transcriptomic data via a variational graph autoencoder, constructing spatially augmented gene expression matrices to improve the accuracy of spatial domain clustering. SpaGCN [13] generates undirected weighted graphs based on spatial distances and histological similarities, using graph convolutions to aggregate gene expression from neighboring spots and identify domains with coherent expression patterns and histological features. CCST [14] employs unsupervised graph convolutional networks (GCNs) to model spatial dependencies, converting spatial data into node-edge graphs (nodes as cells with expression attributes, edges as neighboring relationships) and using multilayer GCNs to enhance clustering by integrating local and global spatial features. GraphST [15] utilizes self-supervised contrastive learning with graph neural networks (GNNs) to embed spatial locations and gene expression into a latent space, enabling the identification of spatial domains by capturing both local proximity and global expression correlations. STAGATE [16] introduces an adaptive graph attention auto-encoder, learning low-dimensional embeddings by integrating spatial and expression data to align spatial domain labels across different datasets—a unique capability for cross-dataset domain analysis. Spatial-MGCN [17] proposes a multi-view graph convolutional network with an attention mechanism, which constructs gene expression and spatial neighbor graphs, extracts unique and shared embeddings, uses a zero-inflated negative binomial decoder for expression matrix reconstruction, and precisely integrates spatial regularization. SpaNCMG [18] proposes a neighborhood-complementary mixed-view graph convolutional network—integrating KNN local or r-radius global info into a complementary graph, adopting attention for adaptive reconstructed expression fusion, and enabling effective marker gene location and unlabeled domain identification.

These methods highlight the critical role of graph structures and multi-modal data integration in addressing the challenge of spatial domain identification, though they face some limitations: SpaGCN and DeepST integrate histological features to enhance clustering but risk introducing noise from image data, complicating accuracy for complex tissues; CCST emphasizes local spatial similarity via GNNs, leading to over-smoothed results and failing to model global semantic structures where distant spots may belong to the same domain; GraphST uses GCN and contrastive learning to improve latent feature learning but struggles with long-range dependencies and lacks multi-level feature integration in contrastive tasks; and STAGATE aligns domains across datasets but cannot correct batch effects, limiting cross-sample consistency.

In spatial transcriptomics, the inherent spatial location information naturally reveals proximity relationships between cells or spots, making graph structures a powerful tool for modeling these spatial correlations. By representing cells/spots as nodes and their spatial adjacency as edges, graphs efficiently capture the spatial dependencies critical for understanding tissue architecture.[12–21] Concurrently, contrastive learning [15,17,18,20,21] has emerged as a key technique to enhance model robustness, particularly in mitigating batch effects and refining latent representations to better distinguish biologically relevant patterns. Additionally, zero-inflated negative binomial (ZINB) models are widely adopted to address the overdispersion and zero-inflated characteristics of transcriptomic data [22,23], providing a biologically grounded framework for accurately modeling gene expression distributions.

**Fig 1:**
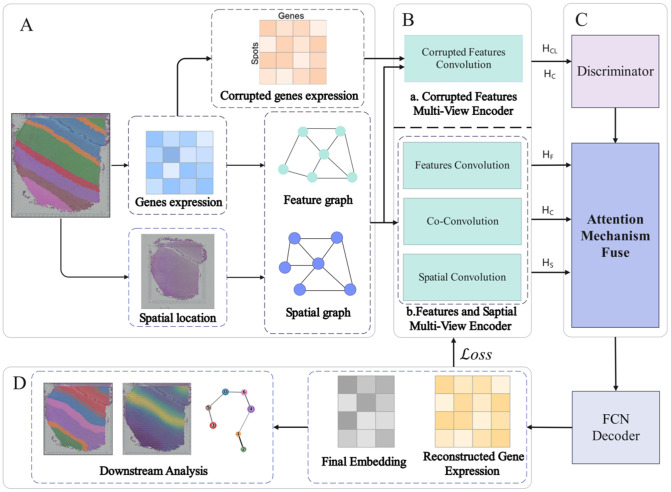
Overview of the DMGCN method. (a) Dataset preprocessing. The preprocessing includes the construction of the spatial graph, based on the Euclidean distance, and the feature graph, based on the Cosine distance. (b) and (c). Multi-view Graph Convolutional (MGCN) encoder. Spatial, feature, and their combination embeddings of both graphs will be generated by the attention mechanism as well, and corrupted features will be trained and discriminated before attention. (d) The result of DMGCN can be used for downstream analysis, such as domain identification and gene expression broadcasting.

Building on these advancements, we introduce DMGCN (Fig 1), a deep-learning approach utilizing a multi-view graph convolutional network enhanced by an attention mechanism and contrastive learning for co-convolution. This method effectively identifies spatial domains and enhances downstream analysis performance. Initially, the preprocessing involves constructing a spatial graph based on Euclidean distance and a feature graph using Cosine distance, capturing spatial position and gene expression in graphical form. Next, a multi-view graph convolutional encoder with an attention mechanism generates embeddings for both graphs, facilitating separate and co-convolution of the spatial and feature graphs, along with the convolution of corrupted features. Finally, the FCN decoder produces domain labels and reconstructs gene expression, which can be utilized in subsequent analyses. The results of DMGCN, including spatial clustering, trajectory inference and other analysis, show the outperformance among all the comparison methods we present in the paper, especially on the 10X Visium HBC dataset.

## Results

### Model performance of domain identification in DLPFC and downstream analysis

**Fig 2:**
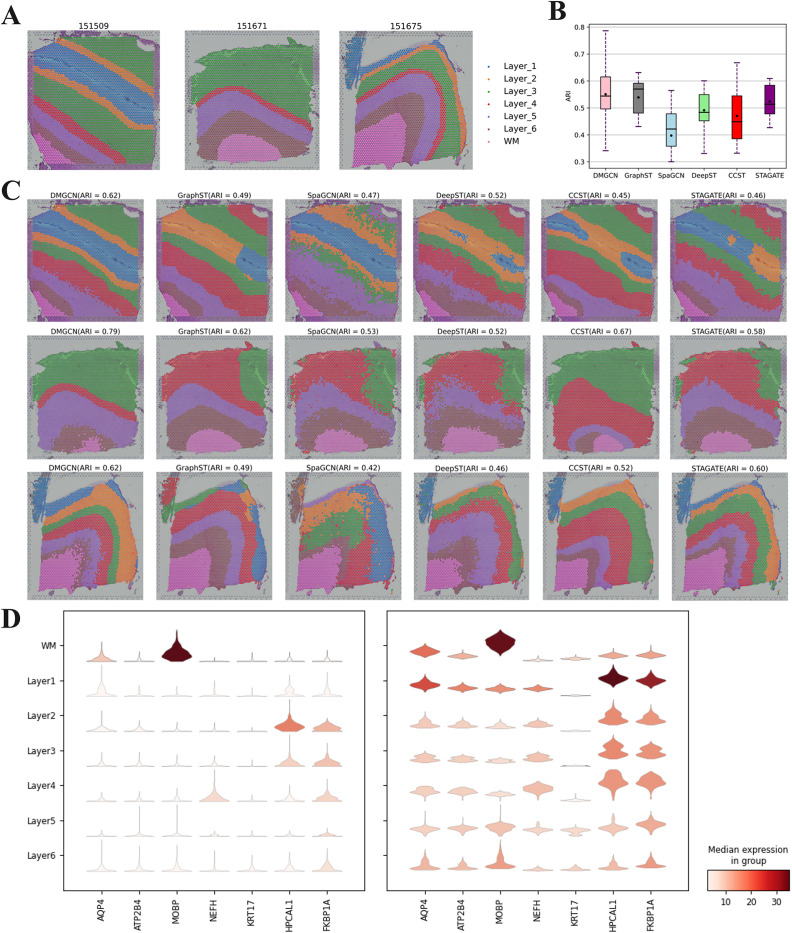
Domain identification results of DMGCN on DLPFC. (a) The manual annotation of 151509, 151671, and 151675 on DLPFC. (b) The ARI boxplots of six methods on all 12 slices of DLPFC. (c) Domain identification of samples 151509, 151671, and 151675 on DLPFC. (d) The marker genes comparison of reconstructed gene expression and raw gene expression.

To evaluate the spatial domain recognition performance of DMGCN, the 10x Visium human dorsolateral prefrontal cortex (DLPFC) dataset [24] proves our model’s capacity in domain identification. Through the experiment on the DLPFC dataset with label information (Fig 2A), the quantitative evaluation, ARI or NMI, was applied to test the algorithm’s performance in handling the complex tissue structures on the specific dataset.

In this experiment, we compared DMGCN with five developed methods that excel in domain identification, allowing us to illustrate the model’s capabilities in comparison. Across all 12 slices, we found that DMGCN achieved the highest performance among all methods on DLPFC (Fig 2B), especially on slices 151509, 151671, and 151675 (Fig 2C).

In addition, compare the violin maps (Fig 2D) between the original gene expression and the reconstructed gene expression, the significant enhancement of these mark genes was found, and this result proves the DMGCN’s capacity to reconstruct the gene expression in the right way.

### Performance of domain identification in HBC and downstream analysis

**Fig 3:**
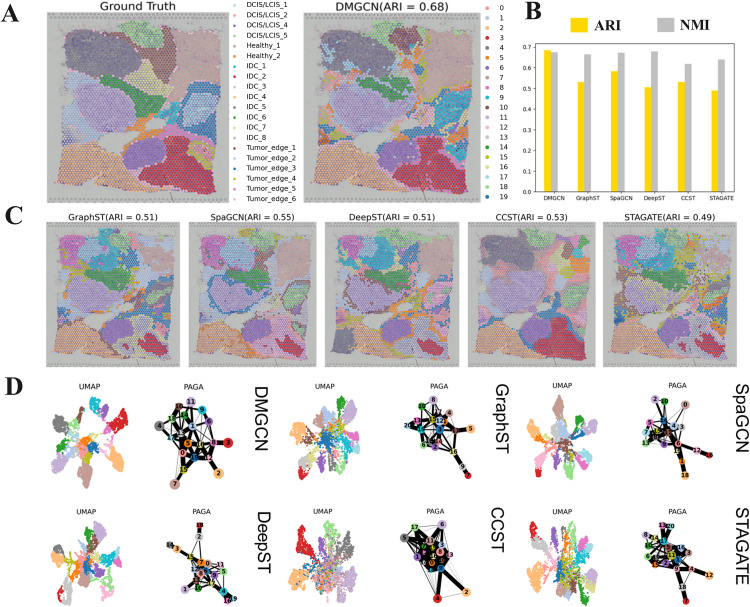
Domain identification results of DMGCN on human breast cancer. (a) The manual annotation of human breast cancer. (b) The ARI histograms of five methods on human breast cancer. (c) Domain identification comparison of human breast cancer. (d) UMAP visualization and PAGA of all methods on human breast cancer.

Following the outstanding performance of the DMGCN model on DLPFC samples, this paper applies the DMGCN model to the human breast cancer dataset for evaluation. In this experiment, we found that, compared to the manual annotation, the results of the DMGCN model, in which the ARI is 0.68 (Fig 3A), show that the DMGCN model has strong accuracy in spatial domain recognition for breast cancer pathology samples and performs better than other methods (Fig 3C), whatever for ARI or NMI (Fig 3B). Except that, the UMAP visualization (Fig 3D) shows that the DMGCN is better at processing the correlation and trajectory of different spatial clusters.

**Fig 4:**
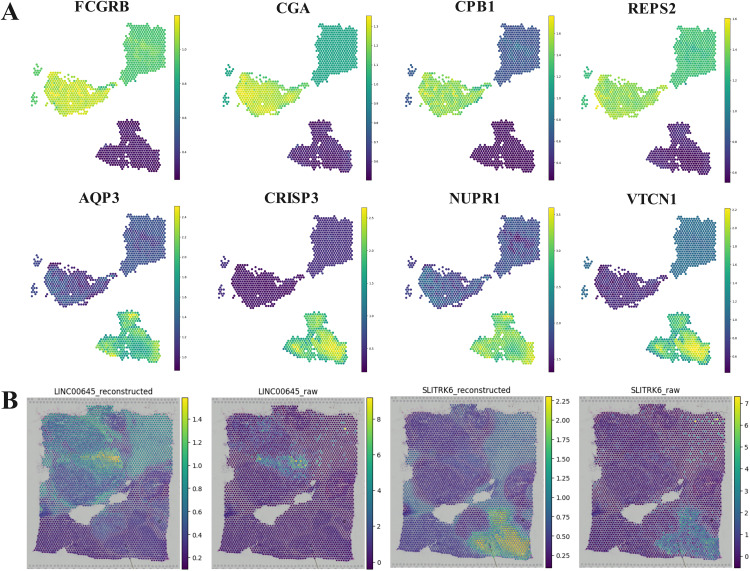
Marker genes analysis. (a) The marker genes of domains 3, 7, and 11 in human breast cancer. (b) The comparison of marker genes, LINC00645 and SLITRK6, in human breast cancer.

Based on the result of DMGCN on human breast cancer, we select domains 3, 7, and 11 that correspond to the IDC_2, Healthy, and IDC_8 in ground truth, and we found that the differentially marker genes [12], such CRISP3, AQP3, VTCN1, NUPR1, CPB1, FCGR3B, CGA, REPS2, can match corresponding pathology areas well (Fig 4A). Except for these marker genes, we still found that the marker genes, LINC00645 and SLITRK6, can be broadcast through the reconstructed gene expression (Fig 4B).

### Performance on Stereo-seq ST data and downstream analysis

**Fig 5:**
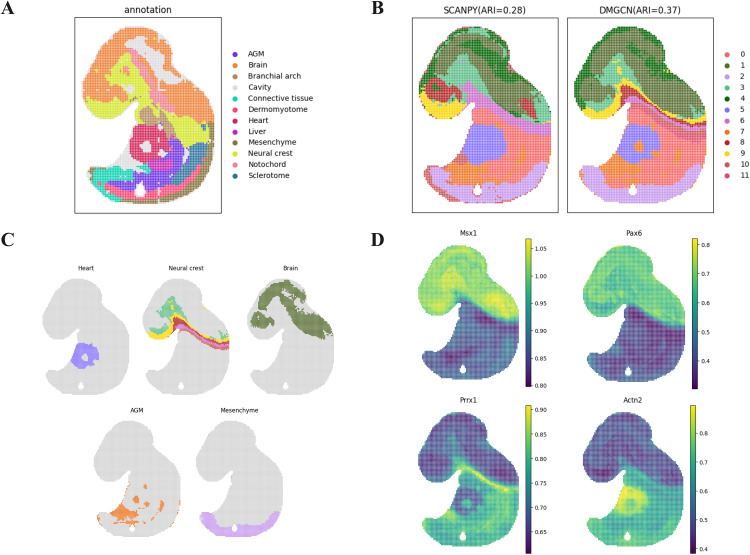
Spatial domain identification on Stereo-seq mouse embryo dataset using DMGCN. (a) Regional annotation of E9.5 mouse embryo tissue in the MOSTA database. (b) The clustering results of SCANPY and DMGCN on the E9.5 mouse embryo dataset. (c) Visualization of partial individual domain identification results by DMGCN (d) Visualization results of corresponding marker gene expression for identified domains on the mouse embryo.

To further assess the generalizability of DMGCN in spatial domain identification, we performed additional validation on a mouse embryo dataset [25] acquired with Stereo-seq technology, complementing the earlier experiments conducted on the 10x Visium platform (Fig 5A). Our results demonstrate that DMGCN effectively captures the laminar structures within the mouse embryo dataset, with 0.37ARI, showing strong alignment with the annotated distributions (Fig 5B). In contrast, the outcomes from SCANPY [26] identified an extensive region enveloping the neural crest and brain, which exhibited limited correspondence with the reference annotations.

In comparison, the segmentation results produced by DMGCN demonstrated stronger agreement with anatomical annotations, both at tissue boundaries and within internal regions. Clusters corresponding to distinct anatomical structures, such as the heart, brain, and liver, were consistently associated with expected marker gene expression patterns (Fig 5C and 5D), thereby validating DMGCN’s localization accuracy.

### Performance on MERFISH data and downstream analysis

We also implemented DMGCN on five adjacent slices of the mouse brain [27] acquired through MERFISH, which include 254 genes labeled across seven distinct structures. For this experiment, we focused on slices 1, 10, and 31 from sample 1, along with slice 112 from sample 3, to compare the performance of spatial domain identification.

**Fig 6:**
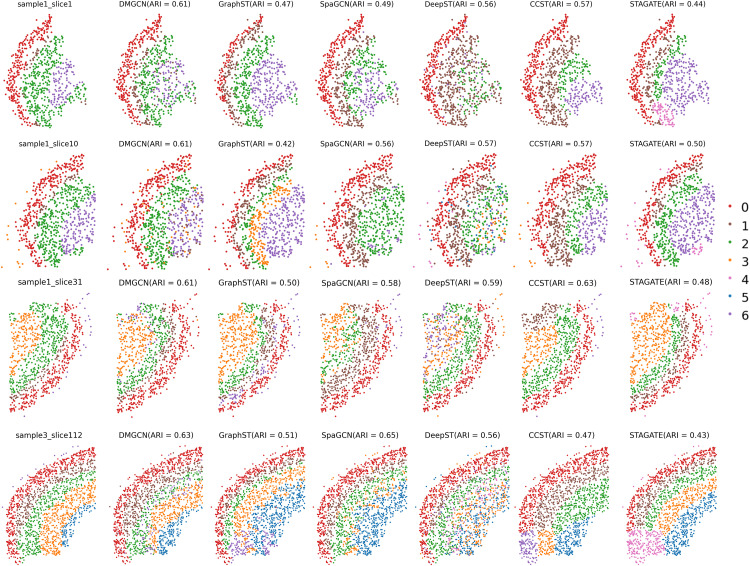
Spatial domain identification on MERFISH mouse brain dataset using DMGCN and other five methods. Compared with ground truth (first column), DMGCN demonstrates higher spatial domain accuracy.

We first examined the domains identified by various methods. Fig 6 illustrates that DMGCN surpasses most other approaches, closely aligning with the manually annotated results. It is important to note that the seven primary spatial domains appear in all four tissue sections, although their shapes and sizes differ across these sections. This pattern proves instrumental for benchmarking the continuity and smoothness of domains generated by different algorithms. Remarkably, DMGCN was able to identify more continuous regions, and when compared to other methods, it nearly achieves the highest performance.

### Ablation Study

To explore how DMGCN operates, we performed a series of ablation studies on the DLPFC dataset. These experiments involved the systematic removal of key components, including the ZINB loss and the Co-convolution loss to evaluate their individual impacts on model performance.

**Fig 7:**
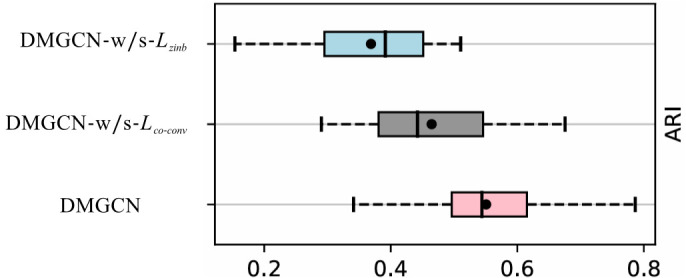
Boxplots of the Adjusted Rand Index for DMGCN and its variants on the DLPFC dataset.

- DMGCN-w/o-$L_{zinb}$: DMGCN has no spatial ZINB loss;
- DMGCN-w/o-$L_{co-conv}$: DMGCN has no Co-convolution loss;

As illustrated in Fig 7, DMGCN consistently surpasses its variants. Notably, DMGCN-w/o-$L_{zinb}$ has the lowest median ARI at just 0.38, the lowest in our ablation tests, highlighting the importance of fitting the gene expression data to the ZINB distribution, which effectively addresses the sparsity of spatial transcriptome data. Conversely, while DMGCN-w/o-$L_{co-conv}$ exhibits the highest variance among all variants, despite achieving a competitive median ARI. This instability implies that consistent and accurate spatial domain identification depends critically on a loss function capable of effectively fusing gene expression and spatial information.

The ablation study further confirms that the integration of multimodal views, spatial and feature, coupled with an attention mechanism, is indispensable to DMGCN’s design. By adaptively learning and combining these complementary information sources, the model achieves not only higher accuracy in domain identification but also significantly improved robustness and stability.

## Conclusion and discussions

In this paper, we propose DMGCN, a multi-view GCN based on contrastive learning for spatial domain identification. DMGCN adopts a multi-view GCN encoder to capture both spatial organization and gene expression similarity, and incorporates an attention mechanism to adaptively learn dependencies between these views. During training the corrupted features are trained in the same way to enhance robustness, while spatial and feature co-convolution are applied to obtain discriminative node embeddings. For the co-convolution embeddings from spatial graph and feature graph, we read out them with graphs and adapt the weights through the discriminator and attention mechanism so that greater weights are given to the significant features. Then, for the K-means clustering and downstream analysis, the final embedding of features and the spatial graph are reconstructed using a decoder, and the reconstructed gene expression will be used to present the heatmap. Furthermore, a zero-inflated negative binomial loss is incorporated into the representation learning to preserve spatial neighborhood information in an end-to-end manner, and the contrastive learning loss is applied to make the positive spots closer and the negative spots further.

Next, we conducted a series of experiments to evaluate the performance of DMGCN. Results from four datasets spanning both 10x Visium and Stereo-seq platforms demonstrate that DMGCN outperforms other comparative methods in accuracy for spatial domain identification. In experiments using the mouse embryo MOSTA dataset, DMGCN successfully distinguishes inner tissue layers. This effectiveness is due to its ability to leverage the relationship between gene expression and spatial information through a multi-view GCN encoder. When analyzing gene spatial expression patterns, the imputed gene expression data from DMGCN more accurately delineate cortical layer boundaries. This enhancement is attributed to the decoder’s ability to model the global probability characteristics of spatial transcriptomic data, resulting in representations that are highly consistent with well-documented gene expression patterns.

Those results highlight the importance of incorporating multi-view representation, attention mechanisms, and contrastive learning within the DMGCN framework. These elements are vital for accurately identifying spatial domains and the effective integration of spatial and feature information. While DMGCN demonstrates strong performance in spatial domain identification, its multi-view GCN encoder requires substantial memory resources, restricting scalability to larger datasets. Future work will focus on enhancing computational efficiency while maintaining accuracy, potentially through distributed training strategies or alternative graph model architectures. In addition, we can further optimize the performance and if possible, the H&E image that includes histological information deserves concern.

## Materials and methods

### Dataset description

We validate the performance of our proposed DMGCN using SRT datasets from three different platforms, which can be downloaded from the public website.

[1] **Human dorsolateral pre-frontal cortex (DLPFC):** The 10x Visium dataset comprises 12 slices, and all slices were manually annotated. The article presents the experimental results of slice 151507, with 4,221 spots and 33,538 genes, and 151674, with 3635 spots and 33,538 genes. The data links to https://www.10xgenomics.com/datasets/visium-hd-cytassist-gene-expression-libraries-of-human-crc.
[2] **Human breast cancer:** The 10x Visium dataset comprises 3,798 spots and 36,601 genes, with 20 regions manually annotated. The data links to https://support.10xgenomics.com/spatial-gene-expression/datasets/1.1.0/V1_Breast_Cancer_Block_A_Section_1.
[3] **Mouse embryo:** The Stereo-seq data comprises 19,527 spots and 27,106 genes, with 12 regions manually annotated for some significant spots. The data links to https://db.cngb.org/stomics/mosta/.
[4] **Mouse brain:** The MERFISH data comprises six samples, with 254 genes and 5–7 regions manually annotated. The samples 1 and 3 were selected to test the performance. The data links to https://doi.brainimagelibrary.org/doi/10.35077/g.21.

### Data preprocessing

During the data processing stage, we first normalize the raw data using the “normalize” function from the SCANPY toolkit. And it’s first to filter genes with 100 “min_cells”, meaning retention of genes expressed in at least 100 cells, and especially, for the MERFISH-seq Mouse Brain dataset, the extra preprocessing is to filter the cells with 100 “min_genes”, meaning retention of cells with at least 100 genes detected, and filter the genes with“min_counts”as 50, for only cells with a total UMI count of ≥1000 were retained.

And then, the highly variable genes will be selected for that model can focus on significant gene information and train effectively. Generally, the 3000 highly variable genes are always a choice for different domain identification methods, and except for the MERFISH-seq Mouse Brain data, it has 254 genes in total, and we set the number of highly variable genes as 30 for training.

Before training, we constructed the spatial proximity map as well as the feature correlation map needed for the multi-view neural network encoder. We used the KNN to obtain the spatial proximity map by calculating the K nearest neighbors of each spatial site based on the Euclidean distance, as well as the feature correlation map by calculating the K nearest neighbors of each gene based on the cosine distance.Finally, we obtained the features graph $A_{f}$, and spatial graph $A_{s}$.And the method of DMGCN to select the K value, and made some adjustments to fit our model, such as the K value of the spatial adjacent graph on the 10X Visium dataset was set as 25, and on the Stereo-seq dataset, we set the K value as 50. Other parameters that are not mentioned here can be found in the S3 Table.

### Data augmentation

For constructing contrastive learning information of the features, the corrupted features $X_{c}\;$is made by randomly shuffling the gene expression and will be trained by the co-convolution with the spatial graph and features graph. The $X_{c}\;$still preserves the topological structure of the original neighborhood graph. Specifically, we generated the contrastive learning label for the calculation of the positive and negative loss after the discriminator.

### Multi-view graph convolutional encoder

The initial integration of genomic and spatial information is achieved by transforming the spatial adjacency graph $A_{s}$ via convolution in the spectral domain, thereby aggregating features from adjacent nodes. Follow Spatial-MGCN [17], this enriched representation is subsequently passed to a multi-layer graph convolutional architecture, which propagates it according to the following hierarchical rule:


$$H_{s}^{\left(l+\text{1}\right)}=ReLU\left({\tilde{D}}_{s}^{-\frac{\text{1}}{\text{2}}}{\tilde{A}}_{s}{\tilde{D}}_{s}^{-\frac{\text{1}}{\text{2}}}H_{s}^{\left(l\right)}W_{s}^{\left(l\right)}\right),$$

where $W_{s}^{\left(l\right)}$ is the weight matrix of layer l in the spatial convolution, initially $H_{s}^{\left(\text{0}\right)}=X$, and ReLU is the activation function. Specifically, we have ${\tilde{A}}_{s}=A_{s}+I$ and ${\tilde{D}}_{s}\;$ is the degree matrix of ${\tilde{A}}_{\text{s}}$.

Applying the same approach used for spatial convolution, we next process feature information by performing feature convolution on the feature association matrix $A_{f}$ and gene expression data:


$$H_{f}^{\left(l+\text{1}\right)}=ReLU\left({\tilde{D}}_{f}^{-\frac{\text{1}}{\text{2}}}{\tilde{A}}_{f}{\tilde{D}}_{f}^{-\frac{\text{1}}{\text{2}}}H_{f}^{\left(l\right)}W_{f}^{\left(l\right)}\right),$$

where $W_{f}^{\left(l\right)}$ is the weight matrix of the lth layer in the feature convolution, initially $H_{f}^{\left(\text{0}\right)}=X$, ${\tilde{A}}_{f}=A_{f}+I$ and ${\tilde{D}}_{f}$ is the degree matrix of ${\tilde{A}}_{f}$.

There is an inherent interconnection between gene expression and spatial localization. When addressing this relationship, we need to account for both their unique attributes and their shared latent features. We introduce a parameter-sharing architecture to train the fused embedding of gene expression and coordinates as Spatial-MGCN [17]. The propagation rule is formulated as follows:


$$H_{sc}^{\left(l+\text{1}\right)}=ReLU\left({\tilde{D}}_{s}^{-\frac{\text{1}}{\text{2}}}{\tilde{A}}_{s}{\tilde{D}}_{s}^{-\frac{\text{1}}{\text{2}}}H_{sc}^{\left(l\right)}W_{c}^{\left(l\right)}\right),$$

$$H_{fc}^{\left(l+\text{1}\right)}=ReLU\left({\tilde{D}}_{f}^{-\frac{\text{1}}{\text{2}}}{\tilde{A}}_{f}{\tilde{D}}_{f}^{-\frac{\text{1}}{\text{2}}}H_{fc}^{\left(l\right)}W_{c}^{\left(l\right)}\right),$$

$$H_{c}^{\left(l+\text{1}\right)}=\;\frac{H_{sc}^{\left(l+\text{1}\right)}+\;H_{fc}^{\left(l+\text{1}\right)}}{\text{2}}\;,$$

where ${\text{W}}_{c}^{\left(\text{l}\right)}$ is the weight matrix of the lth layer in the co-convolution, and $H_{sc}^{\left(l\right)}$ and $H_{fc}^{\left(l\right)}$ are the lth layer embeddings extracted from the spatial and feature maps, respectively, by co-convolution, with the initial $H_{sc}^{\left(\text{0}\right)}$=$H_{fc}^{\left(\text{0}\right)}$=X.

For corrupted features $X_{c}$, the same co-convolution method is used to extract contrastive information from $X_{c}$ to increase the model’s ability to separate positive and negative sample points:


$$H_{cls}^{\left(l+\text{1}\right)}=ReLU\left({\tilde{D}}_{cl}^{-\frac{\text{1}}{\text{2}}}{\tilde{A}}_{s}{\tilde{D}}_{cl}^{-\frac{\text{1}}{\text{2}}}H_{cls}^{\left(l\right)}W_{cl}^{\left(l\right)}\right),$$

$$H_{clf}^{\left(l+\text{1}\right)}=ReLU\left({\tilde{D}}_{cl}^{-\frac{\text{1}}{\text{2}}}{\tilde{A}}_{f}{\tilde{D}}_{cl}^{-\frac{\text{1}}{\text{2}}}H_{clf}^{\left(l\right)}W_{cl}^{\left(l\right)}\right),$$

where $W_{cl}^{\left(l\right)}$ is the weight matrix of the lth layer in the feature convolution, initially $H_{cls}^{\left(\text{0}\right)}=H_{clf}^{\left(\text{0}\right)}=X_{c}$, ${\tilde{A}}_{s}=A_{s}+I$, ${\tilde{A}}_{f}=A_{f}+I$ and, ${\tilde{D}}_{cl}$ is the degree matrix of ${\tilde{A}}_{s}$ and ${\tilde{A}}_{f}$ respectively.

### Attention mechanism

In tasks involving spatial domain identification, the relative contributions of gene expression, spatial context, and their joint representation to the final outcome may vary [17]. To dynamically capture the importance of each latent embedding, we incorporate an attention mechanism that adaptively weights these representations. Specifically, for the joint embedding, we first apply a nonlinear transformation, followed by a Softmax activation to normalize the attention scores across features. The resulting coefficients are computed as follows:


φ=Softmax(W·σ(WH+b)),

where the transformation employs a tanh activation function σ, W denotes a trainable weight matrix and b represents a bias vector. This attention operation is applied to the spatial embedding, feature embedding, and their combined representation to produce an integrated latent embedding:


$$H=\;\varphi_{s}*\;H_{s}+\;\varphi_{f}*\;H_{f}+\;\varphi_{c}*\;H_{c}.$$

### Discriminator

To make the feature embedding of spatial and feature co-convolution more stable and better differentiate between positive and negative sample points, we construct a contrastive learning mechanism between the feature embedding of co-convolution and the feature embedding of corrupted features by using a discriminator to redistribute the weights according to the feature and spatial maps and to generate the contrastive learning labels of the spatial and feature.

Firstly, we processed the co-convolution embedding with the graphs by the average readout for aggregating information of neighboring nodes:


$$W_{s}=\;AvgReadout\left(H_{s},A_{s}\right),$$

$$W_{f}=\;AvgReadout\left(H_{f},A_{f}\right).$$

For the corrupted features, we readout it in the same way:


$$W_{cls}=\;AvgReadout\left(H_{cls},A_{s}\right),$$

$$W_{clf}=\;AvgReadout\left(H_{clf},A_{f}\right).$$

We define the discriminator D(*) to identify the positive and negative spot:


$$D\left(W,Z_{\text{1}},Z_{\text{2}}\right)=\left[BiLinear\left(Z_{\text{1}},W\right),BiLinear\left(Z_{\text{2}},W\right)\right].$$

For the $H_{cls}$ and $H_{clf}$, we discriminate them with $H_{c}\;$respectively and use the attention mechanism to generate the new contrastive learning label:


$$R_{cls}=\varphi_{cls}*\;D\left(W,H_{c},H_{cls}\right)+\;\varphi_{clsa}*\;D\left(W,H_{cls},H_{c}\right),$$

$$R_{clf}=\varphi_{clf}*\;D\left(W,H_{c},H_{clf}\right)+\;\varphi_{clfa}*\;D\left(W,H_{clf},H_{c}\right),$$

where $R_{cls}$ and $R_{clf}$ mean the contrastive learning labels of corrupted features in spatial graph and features graph.

### FCN decoder

The FCN decoder is used to generate the embedding from the result of the attention mechanism and reconstruct the gene expression matrix for the loss calculation and domain identification visualization:


Z=Wz·σ(WH+b),

$$X^{\prime }=Wx\cdot \sigma (WZ+b),$$

where $Z,\;X^{\prime }$ means embedding that used to visualization though K-means and reconstructed gene expression matrix that not only can fit the distribution of features to ZINB distribution.

### Co-Convolution loss

To improve the capability that delineates the positive and the negative points, the discriminator D (*) will be applied to $H_{cls}$, $H_{clf}$,and $H_{c}$, to better constrain the co-convolution that combines with spatial and feature information. The loss of positive and negative points will be calculated by consistency loss for spatial and features co-convolution:


$$L_{con}=∥{\tilde{H}}_{sc}{\tilde{H}}_{sc}^{T}-{\tilde{H}}_{fc}{\tilde{H}}_{fc}^{T}{∥}_{\text{2}}^{\text{2}},$$

and use the binary cross-entropy with logarithmic for the contrastive learning label to calculate the positive loss and negative loss:


$$L_{\text{positive}}=-\frac{\text{1}}{\text{2}N}\left(\sum_{i=\text{1}}^{N}\text{log}\text{D}\left(z_{i},g_{i}\right)+\sum_{i=\text{1}}^{N}\text{log}\left(\text{1}-\text{D}\left(z_{i},g_{i}\right)\right)\right),$$

$$L_{negative}=-\frac{\text{1}}{\text{2}N}\left(\sum_{i=\text{1}}^{N}\text{log}\text{D}\left(z_{i}^{\prime },g_{i}\right)+\sum_{i=\text{1}}^{N}\text{log}\left(\text{1}-\text{D}\left(z_{i}^{\prime },g_{i}\right)\right)\right),$$

where $z_{i},\;z_{i}^{\prime }$ mean the positive and negative samples of the spot i, and $g_{i}$ means the contrastive learning label of spot *i*.

Finally, consistency loss and the summary of positive loss and negative loss will be multiplied with temperature parameter β and γ, respectively:


$$L_{co-conv}=\;\frac{\left(L_{\text{positive}}+\;L_{negative}\right)}{\text{2}}*\;\beta +L_{con}*\;\gamma .$$

### ZINB loss

Based on the observed gene expression data X in spatial transcriptomics, we model its distribution using a ZINB formulation as Spatial-MGCN [17]. Let $\pi_{ij},\nu_{ij},\theta_{ij}$ and $b_{i}$ represent the output parameter matrices of the decoder corresponding to the zero-inflation probability, mean expression, dispersion and biased vectors respectively. The ZINB probability distribution is formally defined as:


$$\begin{array}{cc} p_{zinb}\left(x_{ij}|b_{i}\right) & =ZINB\left(x_{ij}|\pi_{ij},u_{ij},\theta_{ij},b_{i}\right)\\ & =\pi_{ij}\delta_{x_{ij}}=\text{0}+\left(\begin{array}{c} \text{1}-\pi_{i} \end{array}\right)p_{nb}\left(\begin{array}{c} x_{ij}|b_{i} \end{array}\right), \end{array}$$

$$\begin{array}{cc} p_{nb}\left(x_{ij}|b_{i}\right) & =NB\left(x_{ij}|u_{ij},\theta_{ij},b_{i}\right)\\ & =\frac{\Gamma \left(x_{ij}+\theta_{ij}\right)}{\Gamma \left(x_{ij}+\text{1}\right)\Gamma \left(\theta_{ij}\right)}{\left(\frac{\theta_{ij}}{\theta_{ij}+\nu_{ij}}\right)}^{\theta_{i}}{\left(\frac{\nu_{ij}}{\nu_{ij}+\theta_{ij}}\right)}^{x_{ij}}. \end{array}$$

The reconstruction loss for the original gene expression is defined as the negative log-likelihood under the ZINB model, formulated as:

$L_{zinb}=-\frac{\text{1}}{m\left(n_{s}+n_{t}\right)}\sum_{i=\text{1}}^{n_{s}+n_{t}}\;\sum_{j=\text{1}}^{m}\;lnp_{zinb}\left(Z_{ij}|b_{i}\right)$

### Loss function of DMGCN

The training process jointly optimizes the multi-view GCN encoder, the decoder, and the attention mechanism by assigning the proportion of the loss function with certain weights. The final training objective of the model in this paper is defined as:


$$L=\alpha L_{zinb}+L_{co-conv},$$

where α is a temperature parameter that weighs the effects of the ZINB loss.

### Benchmark methods

In our research, we assessed the effectiveness of DMGCN by contrasting it with five prominent methods. These include DeepST [12] and SpaGCN [13], which incorporate gene expression, spatial information, and H&E images; CCST [14], GraphST [15], and STAGATE [16], which integrate gene expression data along with spatial location details; and SCANPY, which focuses exclusively on gene expression profiles. Each approach leverages data with different complexities, making them suitable for the task of domain identification using SRT data.

### Code availability

The code, scripts, and parameter configuration of DMGCN have been shared through GitHub (https://github.com/Jinsl-lab/DMGCN).

## Supporting information

S1 Text Platform and model settings. (DOC)

S1 Table Summary of the datasets used in this study. (XLSX)

S2 Table Memory Usage (MB). (XLSX)

S3 Table Parameter configuration of all datasets. (XLSX)

S4 Table Performance of various loss terms on HBC. (XLSX)

S5 Table Performance of various methods on MERFISH. (XLSX)

S6 Table Performance of various methods on DLPFC. (XLSX)

S7 Table Performance of various methods on HBC. (XLSX)

S1 Fig The spatial domain identification performance of four different parameter combinations on the HBC dataset is shown, among which the combination of term=(1, 1, 10) achieves the highest accuracy. (TIF)

S2 Fig Domain identification results of 151507, 151508, and 151510 on DLPFC. (TIF)

S3 Fig Domain identification results of 151669, 151670, and 151672 on DLPFC. (TIF)

S4 Fig Domain identification results of 151673, 151674, and 151676 on DLPFC. (TIF)

S5 Fig Domain identification results of various methods on MOSTA. (TIF)
