# Supplementary material for: Spatial domain identification method based on multi-view graph convolutional network and contrastive learning
Source: PLoS Comput Biol. 2025 Oct 17;21(10):e1013369. doi: 10.1371/journal.pcbi.1013369 (PMC12533874; doi:10.1371/journal.pcbi.1013369)
Supplement: S1 Text — (DOC) [file pcbi.1013369.s001.doc]

**Supplementary Materials for**

**Spatial domain identification method based on multi-view graph convolutional network and contrastive learning**

Xikeng Liang^1,2, #^, Shutong Xiao^1,2, #^, Lu Ba^1,#^, Yuhui Feng^1,2^, Zhicheng Ma^1,2^, Fatima Adilova^3^, Jing Qi^1,2, *^, Shuilin Jin^1,2, *^

^1^School of Mathematics, Harbin Institute of Technology, Harbin, China

^2^Zhengzhou Research Institute, Harbin Institute of Technology, Zhengzhou, China

^3^ V.I. Romanovsky Institute of Mathematics, Uzbekistan Academy of Sciences, Tashkent, Uzbekistan

^*^ qijing@hit.edu.cn

^#^The authors wish it to be known that, in their opinion, the first three authors should be regarded as Joint First Authors.

1. **Platform and Model Settings**
   1. **Platform and Model Settings**

We implement the DMGCN method on PyTorch 1.11.0, running on an Ubuntu 20.04 server with an Intel(R) Xeon(R) Gold 6258R CPU and an NVIDIA GeForce RTX 4090 GPU.

- 1. **Model Parameters**

The parameters of DMGCN used in each dataset are illustrated in Supplementary Table S3.

The other method’s parameter as follows:

1. GraphST: Refer to the default setting, we set the radius as 50 and the clustering method as ‘Leiden’ for all the datasets, and the resolution searching needs the “increment” and a range between “start” and “end” to fit the fixed cluster numbers, so it wasted times for search automatically, instead of set the fixed resolution to fit the cluster numbers of various data effectively. In these experiments, the “increment”, “start”, and “end” are set as 0.02, 0.1, and 2.5, respectively. Finally, the model learning rate and epochs were 0.001 and 600, with “weight_decay” as 0, and the weighting coefficients of loss (
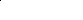
)=(10, 1, 0.1, 10, 1).
2. SpaGCN: Refer to the default setting, the a and b that are used for the adjacent matrix calculation were set to 1 and 49, and the model learning rate and “max_epochs” were 0.005 and 2000 with “weight_decay” as 0. For the resolution searching, “start”, “end”, and “max_run” were set to 0.01, 1000, and 100.
3. DeepST: Refer to the default setting, the “pca_n_comps”, “pre_epochs”, and “epochs” are set as 200, 800, and 1000 in preprocessing. In the training stage, there are too many parameters that are involved in graph construction, augmentation, and clustering to introduce here, and all of them are running in the default setting.
4. CCST: Refer to the default setting, “Dim_PCA” and “min_cells” were set to 5 and 200, and for training, “num_epoch”, “lambda_I”, “DGI”, and “load” were set to 5000, 0.3, 1, and 0. All the datasets are running with data_generation_ST.py and CCST_ST_utils.py, including the MERIFSH-seq dataset, because we have transformed them to .h5ad format as ST datasets.
5. STAGATE: Refer to the default setting, the model learning rate and “n_epochs” were set as 0.0001 and 500, with “weight_decay” as 0.0001 and “gradient_clipping” as 5, and the
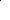
 about the weight of the cell type-aware spatial neighbor network was 0.
