# Supplementary figures and images for: Spatial domain identification method based on multi-view graph convolutional network and contrastive learning
Source: PLoS Comput Biol. 2025 Oct 17;21(10):e1013369. doi: 10.1371/journal.pcbi.1013369 (PMC12533874; doi:10.1371/journal.pcbi.1013369)

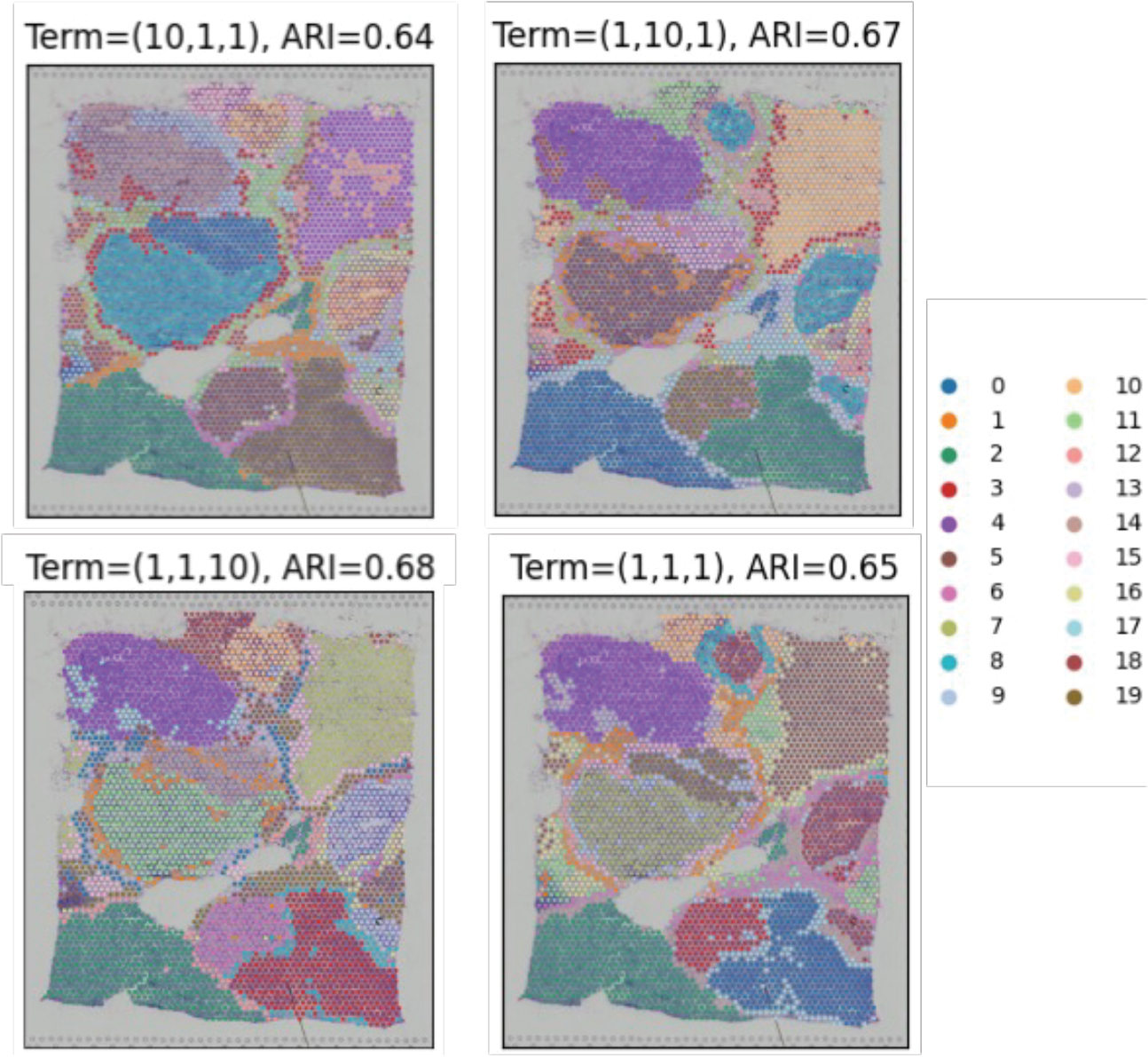

Supplement: S1 Fig — (TIF) [file pcbi.1013369.s009.tif]

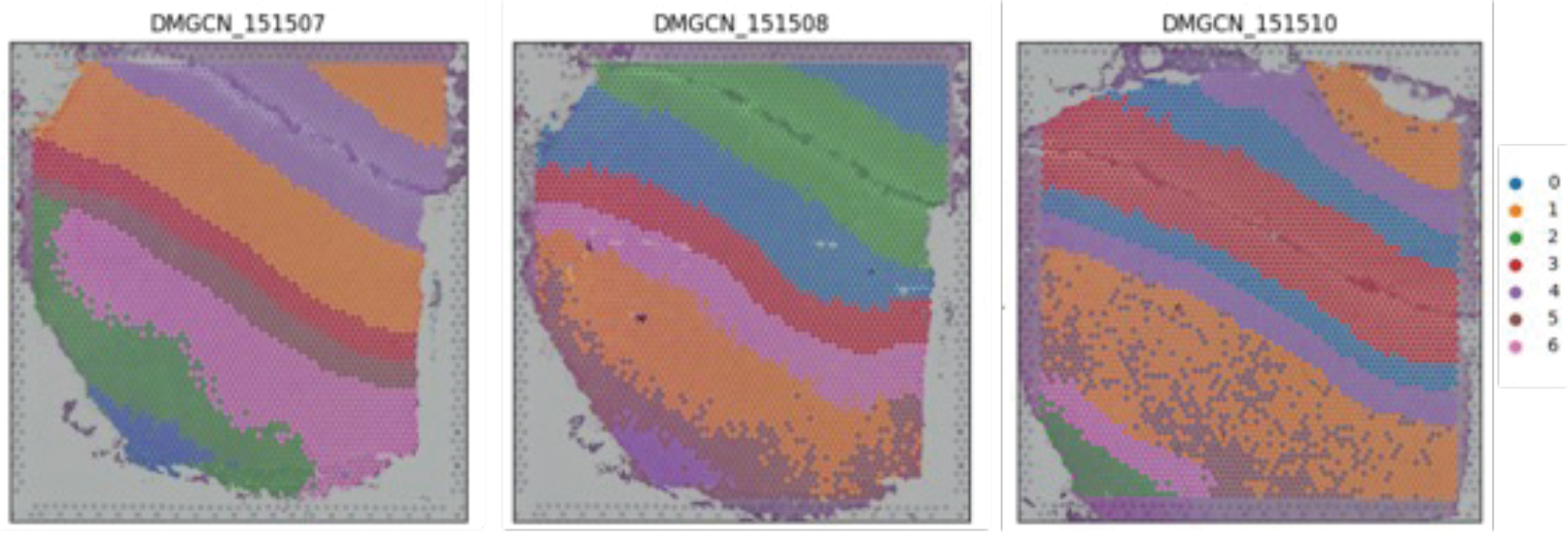

Supplement: S2 Fig — (TIF) [file pcbi.1013369.s010.tif]

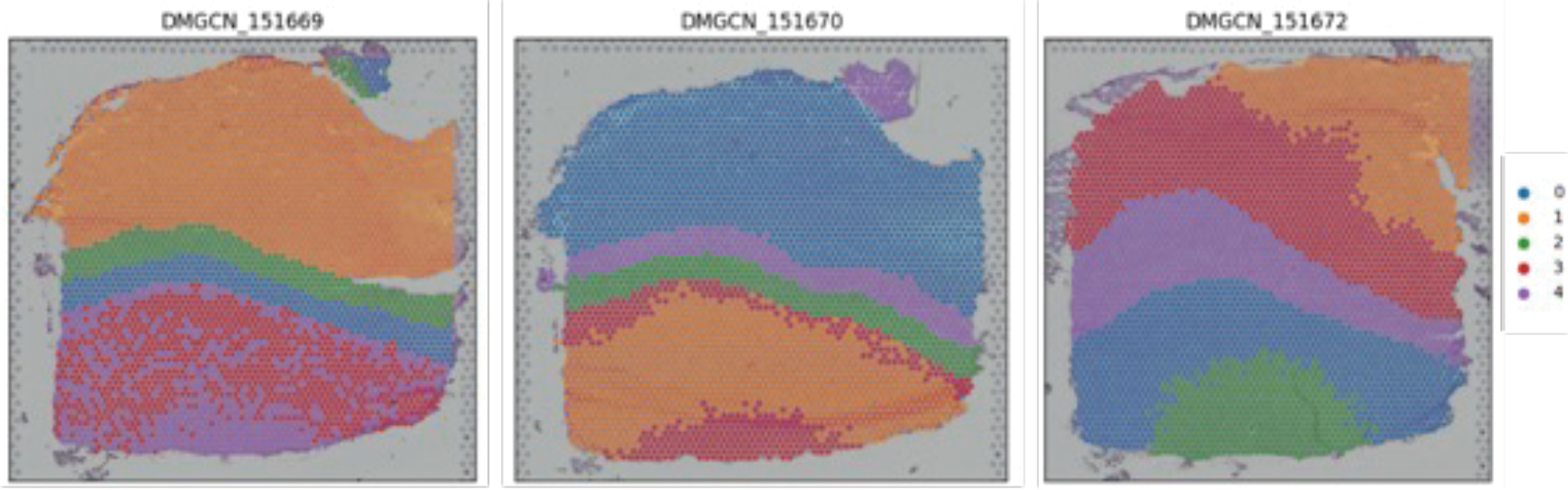

Supplement: S3 Fig — (TIF) [file pcbi.1013369.s011.tif]

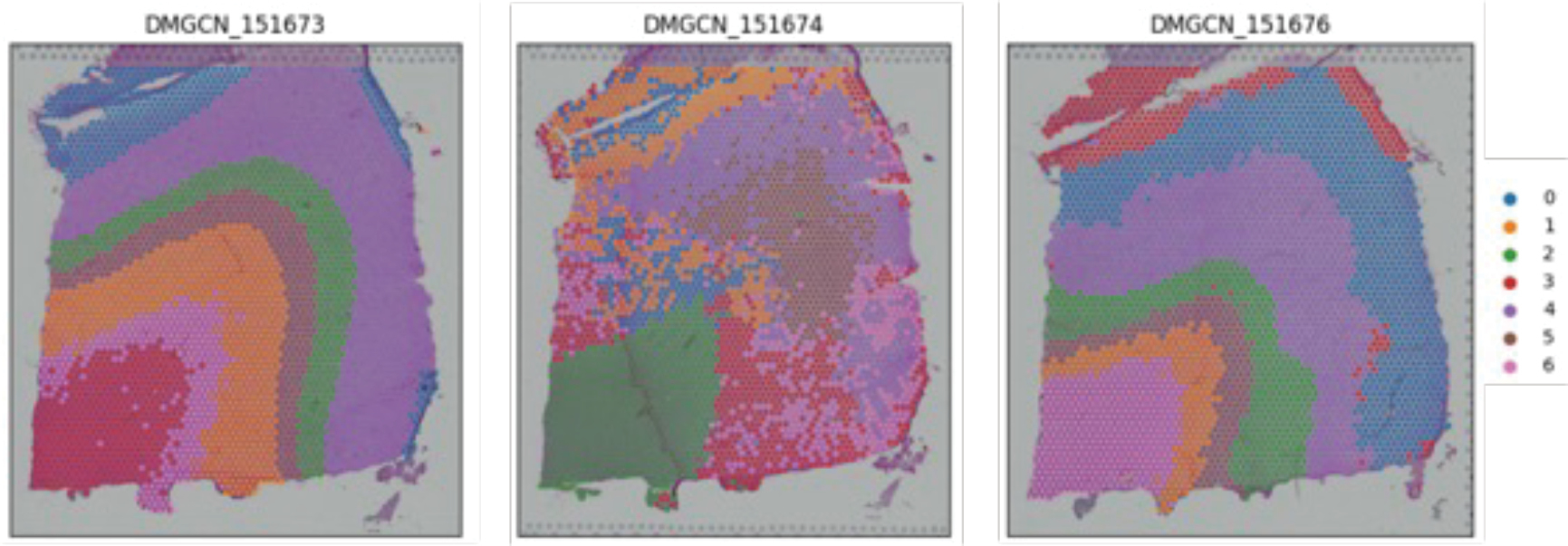

Supplement: S4 Fig — (TIF) [file pcbi.1013369.s012.tif]

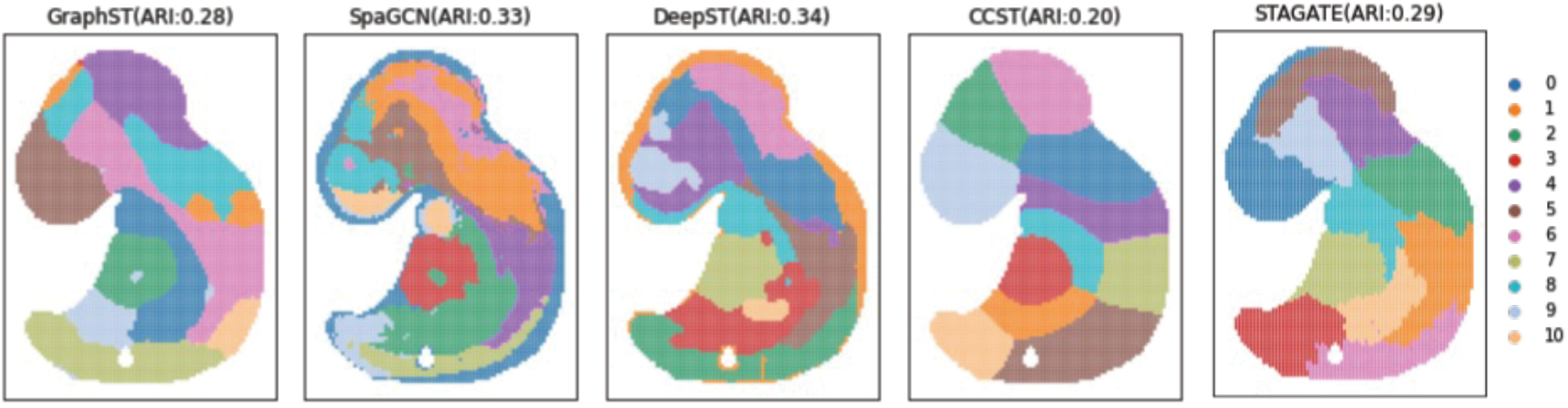

Supplement: S5 Fig — (TIF) [file pcbi.1013369.s013.tif]
